# Histologic features suggesting connective tissue disease in idiopathic pulmonary fibrosis

**DOI:** 10.1038/s41598-020-78140-5

**Published:** 2020-12-03

**Authors:** Ho Cheol Kim, Joon Seon Song, Sojung Park, Hee-Young Yoon, So Yun Lim, Eun Jin Chae, Se Jin Jang, Jin Woo Song

**Affiliations:** 1 Department of Pulmonary and Critical Care Medicine, Asan Medical Center, University of Ulsan College of Medicine, 88 Olympic-ro 43-gil, Songpa-gu, Seoul, 05505 Republic of Korea; 2 Department of Pathology, Asan Medical Center, University of Ulsan College of Medicine, Seoul, Republic of Korea; 3 Department of Radiology, Asan Medical Center, University of Ulsan College of Medicine, Seoul, Republic of Korea

**Keywords:** Connective tissue diseases, Prognostic markers

## Abstract

Some patients with idiopathic pulmonary fibrosis (IPF) have histopathologic features suggesting connective tissue disease (CTD); however, their clinical course and prognosis remain unclear. Thus, we aimed to investigate the clinical course and prognosis of these patients with histologic autoimmune features. Among 114 patients with biopsy-proven IPF, the histologic features were semi-quantitatively graded, and CTD scores (range: 0–9) were calculated as the sum of each score of plasma cell infiltration, lymphoid aggregates, and germinal centres. Patients with high CTD scores (≥ 4) were classified into the interstitial pneumonia with histologic autoimmune features (IP-hAF) group. The mean age of the patients was 60.0 years; 74.6% were men, 69.3% were ever-smokers, and 35.1% had IP-hAF. During follow-up, the IP-hAF group showed slower decline in lung function, and better prognosis (median survival, 48.7 vs. 40.4 months; *p* = 0.015) than the no-IP-hAF group. On multivariate Cox analysis, IP-hAF was an independent prognostic factor (hazard ratio, 0.522; *p* = 0.016), along with the lower diffusing capacity for carbon monoxide, higher scores of reticulation and honeycombing, and usual interstitial pneumonia pattern on high-resolution computed tomography. Patients with IPF having histologic autoimmune features show distinct clinical characteristics and better outcome than those without histologic autoimmune features.

## Introduction

Idiopathic pulmonary fibrosis (IPF) is a chronic progressive fibrosing interstitial pneumonia of unknown cause, characterized by a poor prognosis and median survival of 3 years^1^. The current guideline recommends performing a comprehensive study to exclude connective tissue diseases (CTDs) before diagnosing IPF^2^. However, despite extensive evaluation, some patients with IPF occasionally show clinical, serologic, and/or morphologic features suggesting CTDs, but do not fully satisfy the diagnostic criteria for defined CTDs^3–5^. Recently, the European Respiratory Society (ERS)/American Thoracic Society (ATS) task force devised a new term, interstitial pneumonia with autoimmune features (IPAF), for these disease entities^6^.

Some histopathologic features observed in patients with IPF were reported to suggest the presence of CTD^7–9^. A previous study on 100 patients with the usual interstitial pneumonia (UIP) pattern diagnosed via surgical lung biopsy (39 with CTD and 61 with IPF) showed that patients with CTD-UIP had higher germinal centre and total inflammation scores than those with IPF/UIP, and that a high germinal centre score (odds ratio [OR], 2.948; *p* = 0.001) was an independent discriminating factor for CTD-UIP^7^. Flaherty et al. also showed that in 108 patients with biopsy-proven UIP (99 with IPF and 9 with CTD-UIP), the mean fibroblastic focus score was a significant predicting factor (OR, 8.31; *p* = 0.002) for the presence of IPF-UIP rather than CTD-UIP^8^. In addition, Kono et al. reported that in 111 patients initially diagnosed with IPF, the presence of lymphoid aggregates with germinal centres (hazard ratio [HR], 3.367; *p* < 0.01) was significantly associated with the occurrence of CTD (9.0% of the study population)^9^. Nevertheless, the impact of histopathologic findings suggesting CTD on the clinical course and prognosis of patients with IPF remains unknown.

Therefore, we aimed to investigate the clinical significance of histopathologic features suggesting CTD in patients with IPF. Accordingly, we compared the clinical course and outcomes between patients with IPF having histopathologic autoimmune features and those without histopathologic autoimmune features.

## Material and methods

### Study population

Between February 2000 and November 2007, 187 patients were diagnosed with IPF via surgical lung biopsy at Asan Medical Center, Seoul, Republic of Korea. Among them, 73 patients with unavailable histopathologic slides and/or high-resolution computed tomography (HRCT) images were excluded in this study. All patients underwent thorough systemic history taking, physical examination, and serological testing for CTD at the time of the initial diagnosis, and patients with other known causes of interstitial lung disease (ILD) or those who met the definite diagnostic criteria for CTD were excluded^10–15^. All patients fulfilled the IPF diagnostic criteria of the ATS/ERS/Japanese Respiratory Society/Latin American Thoracic Association^1^, and those with histopathologic features that should raise concerns about the likelihood of not UIP pattern including prominent lymphoplasmacytic infiltration was excluded. Some of the patients in the present study had been included in a previous study^7^. This study was approved by the Institutional Review Board of Asan Medical Center (2014-0911). The requirement for informed consent was waived by the Institutional Review Board of Asan Medical Center, because of the retrospective nature of the study. All methods were performed in accordance with the Declaration of Helsinki and the relevant guidelines.

### Data collection

Clinical and survival data of all patients were obtained from medical records, telephonic interviews, and/or the records of the National Health Insurance Service of Korea. All clinical parameters were obtained within 1 month before surgical lung biopsy. Spirometry, diffusing capacity of the lung for carbon monoxide (DLco), and total lung capacity (TLC) determined using plethysmography were measured according to the ERS/ATS recommendations^16,17^.

### Pathologic evaluation

The pathologic slides were reviewed by two thoracic pathologists (J.S.S. and S.J.J.) blinded to the clinico-radiologic information. The following histologic characteristics were semi-quantitatively graded according to a previously described grading system with a slight modification and presented in Supplementary Material Figs. 1 to 7^7^: plasma cell infiltration, lymphoid aggregates, the overall extent of mononuclear cell interstitial inflammation (total inflammation), organizing pneumonia, fibroblastic foci, intra-alveolar macrophages, and stromal fibrosis (all scored between 0 and 3), as well as honeycombing and pleural changes. Honeycombing was scored according to the measured size of the largest honeycombing spaces in the biopsy specimen: score 0, none; 1, < 1 mm; 2, 1–3 mm; 3, 3–5 mm; and 4, > 5 mm. Pleural changes were scored according to the level of inflammatory change of the pleura: score 1, normal; 2, pleural fibrosis; 3, fibrinous pleuritis, and 4, fibrosis and fibrinous pleuritis. We counted the number of germinal centres on three independent low-power fields (4×) using light microscopy. Thereafter, germinal centres were scored according to the average number of germinal centres in these three areas: 0, none; 1, < 5; 2, ≥ 5 but < 10; and 3, ≥ 10. The presence of perivascular collagen was also investigated. Disagreement (≥ 2 grades) between the two pathologists was resolved via consensus.

Of these pathologic parameters, the scores of plasma cell infiltration, lymphoid aggregates, and germinal centres were considered to represent distinctive histologic features of CTD-ILD^7,18^, and their sum was defined as the CTD score.

### Radiologic evaluation

The HRCT images acquired at the time of diagnosis were reviewed by two thoracic radiologists (S.Y.L. and E.J.C.) in a blinded manner. The extent of reticular abnormality, emphysema, ground-glass opacity, traction bronchiectasis, consolidation, and honeycombing were semi-quantitatively scored on a scale of 25% for all lobes (0- to 4-point scales). Radiologic features associated with non-specific interstitial pneumonia (NSIP) or CTD, such as the presence of peribronchovascular distribution, subpleural sparing, pleural or pericardial effusion, and oesophageal dilatation, were also evaluated^19,20^. Overall, the HRCT pattern was categorised as UIP or non-UIP^1^. The UIP pattern was defined as a subpleural, basal predominance of reticular abnormalities, honeycombing with or without traction bronchiectasis, and the absence of findings inconsistent with a UIP pattern including extensive ground-glass opacity, micronodules, discrete cysts, mosaic attenuation, or segmental/lobar consolidation^1^. Disagreement between the two readers was resolved via consensus.

### Statistical analysis

All values were presented as mean ± standard deviation for continuous variables or as percentages for categorical variables. Student’s t-test or Mann–Whitney U test was used for continuous data, and Pearson’s chi-square test or Fisher’s exact test was used for categorical data. Receiver operating characteristic (ROC) curve analysis was performed to confirm the optimal cut-off value of CTD scores for predicting mortality in the study population. Changes in lung function were evaluated using a paired t-test for intragroup comparison and Student’s t-test for intergroup comparison. Survival was assessed using Kaplan–Meier survival curves, and the differences between groups were evaluated using the log-rank test. To identify variables that predicted histologic autoimmune features or survival, we used logistic or Cox regression analysis with backward elimination; variables with *p* < 0.2 in the univariate analysis were entered into the multivariate models. All statistical analyses were performed using IBM SPSS Statistics for Windows/Macintosh, Version 23.0 (IBM Corp., Armonk, USA). A value of *p* < 0.05 was considered significant (two-tailed).

## Results

### Study population

The median follow-up period was 40 months. The mean age of the patients was 60.0 years; 74.6% were men, and 69.3% were ever-smokers (Table 1). During follow-up, 80 (70.2%) patients died. and no significant difference was observed in treatment between survivors and non-survivors. Non-survivors had lower lung function (forced vital capacity [FVC] and TLC) than survivors (Table 1). Non-survivors also had higher scores of reticulation and honeycombing; lesser frequent subpleural sparing; more frequent UIP pattern on HRCT; and lower lymphoid aggregate, and CTD scores on surgical lung biopsy than survivors (Tables 2, 3).

**Table 1:** Comparison of the baseline characteristics between non-survivors and survivors among patients with IPF.

	Total	Non-survivors	Survivors	*p* value
Patient number	114	80	34	
Age, years	60.0 ± 7.0	59.9 ± 7.5	60.3 ± 5.8	0.759
Female sex	29 (25.4)	18 (22.5)	11 (32.4)	0.269
Ever-smoker	79 (69.3)	59 (73.8)	20 (58.8)	0.114
ANA positivity	33 (30.0)	22 (28.6)	11 (33.3)	0.617
RF positivity	13 (12.0)	11 (14.5)	2 (6.3)	0.337
Other autoantibody*	4 (3.5)	3 (3.8)	1 (2.9)	> 0.999
CRP, mg/L	0.4 ± 0.4	0.5 ± 0.4	0.3 ± 0.5	0.141
FVC, % predicted	74.3 ± 16.8	71.9 ± 16.2	80.0 ± 17.2	0.019
DLco, % predicted	66.9 ± 18.0	65.1 ± 18.1	71.1 ± 17.2	0.106
TLC, % predicted	73.6 ± 15.3	71.4 ± 15.1	77.9 ± 15.1	0.042
BAL fluid, % (n = 66)				
Neutrophils	8.7 ± 9.5	9.5 ± 10.4	6.6 ± 6.1	0.260
Lymphocytes	16.9 ± 13.2	17.0 ± 13.1	16.5 ± 13.9	0.888
Treatment with steroids and/or cytotoxic agents**	96 (84.2)	69 (86.3)	27 (79.4)	0.360

Data are presented as mean ± standard deviation, number (%), or median (interquartile range), unless otherwise indicated.

IPF, idiopathic pulmonary fibrosis; ANA, anti-nuclear antibody; RF, rheumatoid factor; CRP, C-reactive protein; FVC, forced vital capacity; DLco, diffusing capacity of the lung for carbon monoxide; TLC, total lung capacity; BAL, bronchoalveolar lavage.

*Other autoantibody included anti-Ro (n = 1) and anti-Scl70 antibody (n = 3).

**Cytotoxic agents included azathioprine, cyclophosphamide, cyclosporine, and mycophenolate mofetil.

**Table 2:** Comparison of high-resolution computed tomography images between non-survivors and survivors among patients with IPF.

Characteristic	Total	Non-survivors	Survivors	*p* value
Patients, number	114	80	34	
Reticulation	1.3 ± 0.6	1.4 ± 0.7	1.1 ± 0.3	0.001
Honeycombing	0.7 ± 0.7	0.9 ± 0.7	0.3 ± 3.5	< 0.001
Ground-glass opacity	0.8 ± 0.8	0.7 ± 0.8	0.9 ± 0.9	0.282
Consolidation	0.2 ± 0.4	0.2 ± 0.4	0.1 ± 0.3	0.432
Emphysema	0.5 ± 0.6	0.5 ± 0.6	0.5 ± 0.7	0.541
Traction bronchiectasis	2.5 ± 0.9	2.6 ± 0.8	2.2 ± 1.0	0.059
Peribronchovascular distribution	41 (36.3)	27 (34.2)	14 (41.2)	0.478
Subpleural sparing	11 (9.7)	4 (5.1)	7 (20.6)	0.017
Pleural or pericardial effusion	3 (2.7)	2 (2.5)	1 (2.9)	> 0.999
Esophageal dilatation	5 (4.4)	5 (6.3)	0	0.320
UIP pattern	79 (69.3)	65 (81.3)	14 (41.2)	< 0.001

Data are presented as mean ± standard deviation or number (%), unless otherwise indicated.

IPF, idiopathic pulmonary fibrosis; UIP, usual interstitial pneumonia; CTD, connective tissue disease.

**Table 3:** Comparison of histologic findings between non-survivors and survivors among patients with IPF.

Characteristic	Total	Non-survivors	Survivors	*p* value
Patients, number	114	80	34	
Fibroblastic foci	1.5 ± 0.7	1.6 ± 0.8	1.3 ± 0.6	0.076
Lymphoid aggregates	1.5 ± 0.7	1.5 ± 0.7	1.8 ± 0.7	0.029
Plasma cell infiltration	1.0 ± 0.8	0.9 ± 0.7	1.2 ± 0.9	0.107
Germinal centres	0.5 ± 0.8	0.4 ± 0.8	0.7 ± 1.0	0.078
Total inflammation	1.6 ± 0.6	1.6 ± 0.6	1.8 ± 0.6	0.071
Pleural change	2.5 ± 0.9	2.4 ± 1.0	2.6 ± 0.8	0.398
Organizing pneumonia	0.7 ± 0.8	0.7 ± 0.8	0.7 ± 0.7	0.826
Intra-alveolar macrophages	1.2 ± 0.6	1.2 ± 0.6	1.2 ± 0.7	0.646
Honeycombing	2.5 ± 1.2	2.6 ± 1.2	2.4 ± 1.1	0.420
Stromal fibrosis	1.8 ± 0.8	1.8 ± 0.8	1.7 ± 0.8	0.722
Perivascular collagen	0.1 ± 0.3	0.1 ± 0.3	0.1 ± 0.3	0.914
CTD score	3.0 ± 1.9	2.7 ± 1.7	3.6 ± 2.1	0.017

Data are presented as mean ± standard deviation or number (%), unless otherwise indicated.

IPF, idiopathic pulmonary fibrosis; UIP, usual interstitial pneumonia; CTD, connective tissue disease.

### Prognostic factors in IPF

On univariate Cox analysis, lower lung function (FVC, DLco, and TLC), poorer exercise capacity (distance and the lowest oxygen saturation during the 6MWT), higher scores of reticulation and honeycombing, and a UIP pattern on HRCT were significant predictors of mortality (Supplementary Material Table 1). In addition, higher fibroblastic focus scores and lower germinal centre scores were significant prognostic factors. On multivariate Cox analysis, lower CTD scores were independently associated with an increased risk of mortality (HR, 0.837; 95% confidence interval [CI], 0.724–0.969; *p* = 0.017) in patients with IPF, along with lower DLco, higher scores of reticulation and honeycombing, and a UIP pattern on HRCT (Table 4). On ROC analysis, the optimal cut-off level of CTD scores for predicting mortality in patients with IPF was 3.5 (sensitivity, 72.5%; specificity, 52.9%; *p* = 0.024), and patients with high CTD scores (≥ 4) were classified into the interstitial pneumonia with histologic autoimmune features (IP-hAF) group.

**Table 4:** Predicting factors for survival in patients with IPF assessed using a multivariate Cox hazard model including CTD score.

Variable	HR (95% CI)	*p* value
DLco	0.982 (0.967–0.998)	0.025
Reticulation	2.372 (1.600–3.518)	< 0.001
Honeycombing	1.837 (1.290–2.617)	0.001
UIP pattern on HRCT	2.025 (1.096–3.743)	0.024
CTD score	0.837 (0.724–0.969)	0.017

IPF, idiopathic pulmonary fibrosis; HR, hazard ratio; CI, confidential interval; DLco, diffusing capacity of the lung for carbon monoxide; UIP, usual interstitial pneumonia; HRCT, high-resolution computed tomography; CTD, connective tissue disease.

Total lung capacity (r = 0.877; *p* < 0.001) was not included in the Cox proportional hazard model because of its high correlation with forced vital capacity (FVC).

### Baseline characteristics

Histopathologic findings of typical IP-hAF patients were shown in Supplementary Material Fig. 8. The IP-hAF group included 35.1% of patients, and comprised more women and never-smokers than the no-IP-hAF group (Table 5). Additionally, the IP-hAF group showed a tendency of lower DLco than the no-IP-hAF group. However, no significant differences were observed in radiologic parameters between the groups except for higher consolidation scores (0.3 vs. 0.1; *p* = 0.079) and more frequent presence of peribronchovascular distribution (47.5% vs. 30.1%; *p* = 0.066) in the IP-hAF group than in the no-IP-hAF group (Supplementary Material Table 2). The IP-hAF group had higher scores of lymphoid aggregates (2.3 vs. 1.2; *p* < 0.001), plasma cell infiltration (1.8 vs. 0.5; *p* < 0.001), germinal centres (1.1 vs. 0.1; *p* < 0.001), total inflammation (2.2 vs. 1.3; *p* < 0.001), pleural changes (2.8 vs. 2.2; *p* = 0.001), perivascular collagen (0.2 vs. 0.1; *p* = 0.014), and CTD score (5.1 vs. 1.8; *p* < 0.001) than the no-IP-hAF group (Supplementary Material Table 3). The IP-hAF group also had lower scores of honeycombing (2.2 vs. 2.7; *p* = 0.016) and stromal fibrosis (1.5 vs. 1.9; *p* = 0.012) than the no-IP-hAF group.

**Table 5:** Comparison of the baseline characteristics between the IP-hAF group and no-IP-hAF group among patients with IPF.

Feature	IP-hAF	no-IP-hAF	*p* value
Patient number	40	74	
Age, years	59.6 ± 7.1	60.2 ± 7.0	0.670
Female sex	19 (47.5)	10 (13.5)	< 0.001
Ever-smoker	20 (50.0)	59 (79.7)	0.001
ANA positivity	15 (38.5)	18 (25.4)	0.151
RF positivity	6 (16.2)	7 (9.9)	0.362
CRP, mg/L	0.5 ± 0.6	0.4 ± 0.3	0.478
FVC, % predicted	71.0 ± 16.9	76.2 ± 16.7	0.124
DLco, % predicted	62.7 ± 14.8	69.3 ± 19.2	0.064
TLC, % predicted	71.2 ± 14.4	74.8 ± 15.7	0.262
BAL fluid, % (n = 66)			
Neutrophils	8.7 ± 7.7	8.8 ± 10.3	0.977
Lymphocytes	14.6 ± 11.4	18.0 ± 14.1	0.336
Treatment with steroids and/or cytotoxic agents*	33 (82.5)	63 (85.1)	0.713

Data are presented as mean ± standard deviation or number (%), unless otherwise indicated.

IP-hAF, interstitial pneumonia with histologic autoimmune features; IPF, idiopathic pulmonary fibrosis; ANA, anti-nuclear antibody; RF, rheumatoid factor; CRP, C-reactive protein; FVC, forced vital capacity; DLco, diffusing capacity of the lung for carbon monoxide; TLC, total lung capacity; BAL, bronchoalveolar lavage.

*Cytotoxic agents included azathioprine, cyclophosphamide, cyclosporine, and mycophenolate mofetil.

On univariate logistic analysis, female sex and never-smoking were significantly associated with the IP-hAF group (Supplementary Material Table 4). However, on multivariate logistic analysis, female sex (OR, 6.624; 95% CI, 2.548–17.222; *p* < 0.001) and higher scores of consolidation (OR, 4.072; 95% CI, 1.293–12.823; *p* = 0.016) were independently associated with the IP-hAF group.

### Changes in lung function

**Figure 1:**
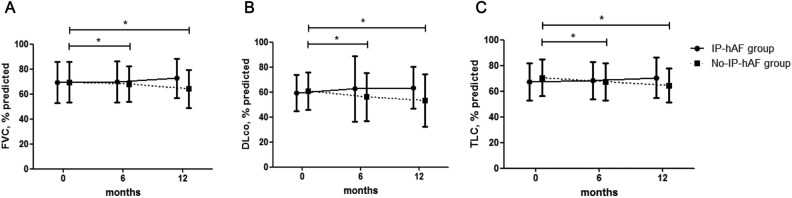
Comparison of changes in lung function between the interstitial pneumonia with histologic autoimmune features (IP-hAF) group and no-IP-hAF group among patients with idiopathic pulmonary fibrosis (IPF). (**a**) Changes in FVC. (**b**) Changes in DLco. (**c**) Changes in TLC. Each symbol with error bars represents the mean and standard deviation. * means *p* < 0.05. FVC, forced vital capacity; DLco, diffusing capacity of the lung for carbon monoxide; TLC, total lung capacity.

Among all patients (n = 114), 96 (84.2%) were treated with steroids and/or immunosuppressants, and the proportion of treated patients did not differ between the IP-hAF and no-IP-hAF groups (Table 5). During follow-up, the IP-hAF group did not show any decline in lung function (FVC, DLco, and TLC); however, the no-IP-hAF group showed a significant decline for 6 or 12 months after diagnosis (Fig. 1, Supplementary Material Table 5). A comparison of the decline in lung function revealed that the no-IP-hAF group had a greater decline in DLco after 6 months and in FVC, DLco, and TLC after 12 months than the IP-hAF group (Supplementary Material Table 5).

When stratified according to treatment, the IP-hAF group did not show any decline in lung function regardless of treatment (Supplementary Material Fig. 9a–c); however, in the no-IP-hAF group, the treatment group showed a significant decline in FVC, DLco, and TLC for 6 or 12 months after diagnosis, and the no-treatment group showed only a significant decline in FVC for 12 months after diagnosis (Supplementary Material Fig. 9d–f). A comparison of the decline in lung function revealed no significant difference between the treatment and non-treatment groups (Supplementary Material Table 6).

### Clinical course and outcome

**Figure 2:**
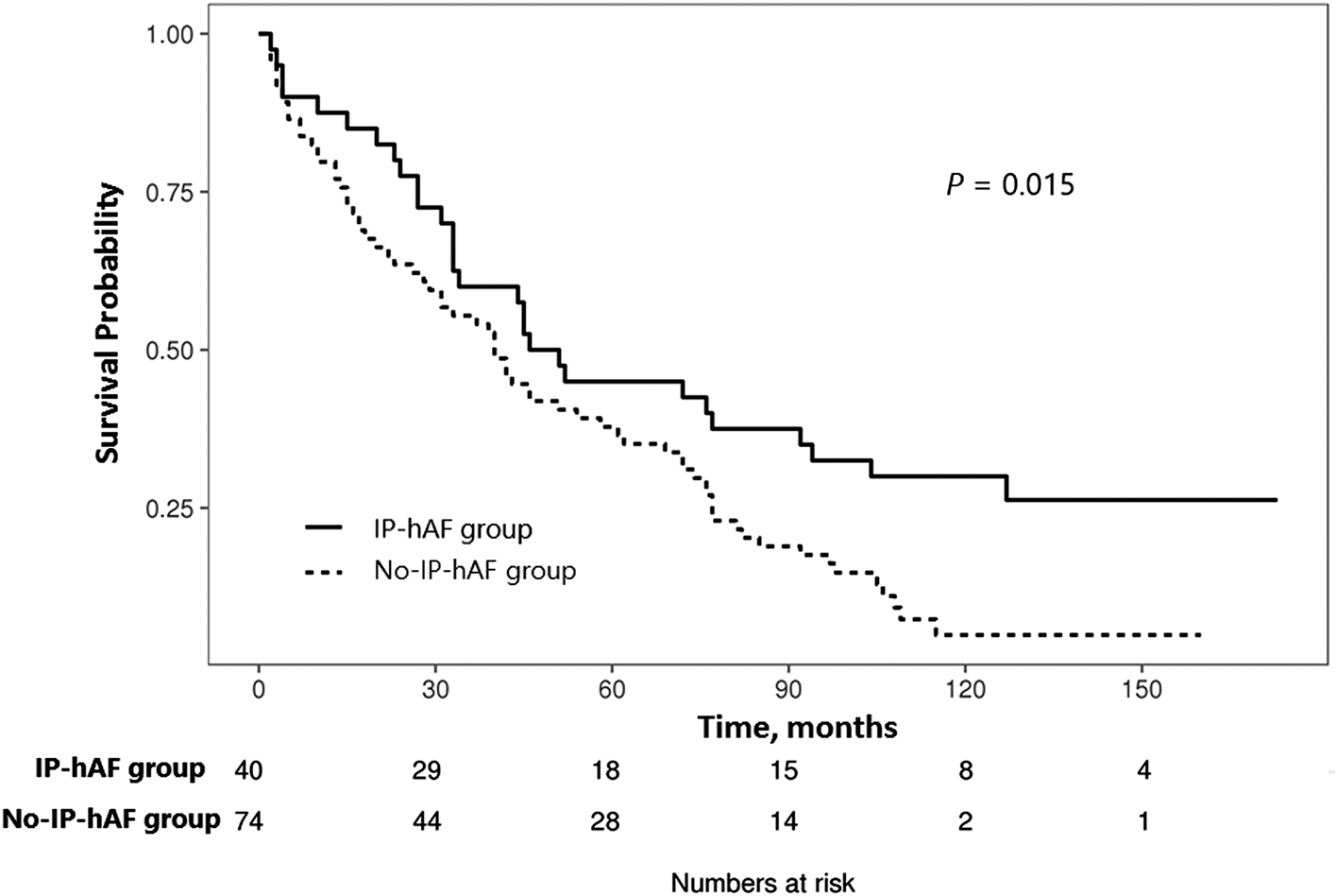
Comparison of survival curves between the interstitial pneumonia with histologic autoimmune features (IP-hAF) group and no-IP-hAF group among patients with idiopathic pulmonary fibrosis (IPF).

During follow-up, CTDs occurred in 2 (5%) patients (1 rheumatoid arthritis and 1 Sjogren syndrome) in the IP-hAF group; however, none occurred in the no-IP-hAF group (*p* = 0.121). The IP-hAF group showed better survival (median survival, 48.7 vs. 40.4 months; *p* = 0.015) than the no-IP-hAF group (Fig. 2). Likewise CTD score, IP-hAF was also an independent prognostic factor (HR, 0.522; 95% CI, 0.307–0.886; *p* = 0.016) in patients with IPF, along with lower DLco, higher scores of reticulation and honeycombing, and a UIP pattern on HRCT, as seen on multivariate Cox analysis (Table 6, Supplementary Material Table 7).

**Table 6:** Predicting factors for survival in patients with IPF assessed using a multivariate Cox hazard model including diagnosis of IP-hAF.

Variable	HR (95% CI)	*p* value
DLco	0.985 (0.970–0.999)	0.043
Reticulation	2.453 (1.656–3.631)	< 0.001
Honeycombing	1.767 (1.242–2.515)	0.002
UIP pattern on HRCT	1.966 (1.088–3.552)	0.025
IP-hAF group	0.522 (0.307–0.886)	0.016

IPF, idiopathic pulmonary fibrosis; HR, hazard ratio; CI, confidential interval; DLco, diffusing capacity of the lung for carbon monoxide; UIP, usual interstitial pneumonia; HRCT, high-resolution computed tomography; IP-hAF, interstitial pneumonia with histologic autoimmune features.

Total lung capacity (r = 0.877; *p* < 0.001) was not included in the Cox proportional hazard model because of its high correlation with forced vital capacity (FVC).

## Discussion

This study revealed significant histologic autoimmune features in one-third of those in the IPF cohort. The IP-hAF group included patients who were more frequently women and never-smokers, had more stable lung function and frequent CTD development, and had better survival than the no-IP-hAF group. IP-hAF was an independent risk factor for mortality in patients with IPF.

Some histologic features, such as lymphoplasmacytic infiltration and lymphoid aggregates with or without germinal centres, have been reported to suggest CTD^7,21^. A previous study on 100 patients with a UIP pattern diagnosed via surgical lung biopsy (39 with CTD and 61 with IPF) reported that patients with CTD-UIP had higher total inflammation and germinal centre scores than those with IPF/UIP^7^. Ozasa et al. studied 105 patients with idiopathic interstitial pneumonia (IIP; 79 with UIP and 26 with NSIP) and 49 with CTD-ILD and showed that plasma cell infiltration, lymphoid follicle with germinal centres, and airspace fibrin were predictive of CTD-ILD^21^. On the basis of these results, lymphoplasmacytic infiltration and interstitial lymphoid aggregates with germinal centres were suggested as histopathologic patterns of the morphologic domain in IPAF^6^. In our study, one-third of patients with IPF showed these findings. Our findings are supported by a previous study by Omote et al.^22^ on 44 patients with IIP and autoantibodies (25 with UIP, 13 with NSIP, and 6 with other histologic patterns). They reported observing two or more characteristic histologic features suggesting CTD (e.g. prominent plasmacytic infiltration and lymphoid aggregates with germinal centres) in 60% of patients with histologic UIP^22^.

Our results showed that female sex and consolidation on HRCT were independent predicting factors of IP-hAF. Previous studies also reported female predominance in IPAF cohorts (52.1–71.4%)^23–26^. Alhamad et al. studied 118 patients (61 with IPF, 29 with CTD-UIP, and 28 with lung-dominant CTD-UIP) and reported that patients with CTD-UIP and lung-dominant CTD-UIP were more likely to be female than patients with IPF (72.4% with CTD-UIP, 67.9% with lung-dominant CTD-UIP, and 47.5% with IPF; *p* = 0.042)^27^. The radiologic organizing pneumonia pattern, characterized by consolidation, is considered a morphologic domain in the IPAF criteria^6^. Yoshimura et al. studied 194 patients with chronic fibrosing interstitial pneumonia (163 with IPF and 31 with NSIP) and reported more frequent NSIP with a combined organizing pneumonia pattern (12.5 vs. 4.3%) on HRCT in the IPAF group than in the non-IPAF group^28^.

In our study, lung function declines over 12 months were slower in the IP-hAF group than in the no-IP-hAF group. Our findings were consistent with those of previous reports^22,29,30^. Omote et al. studied 23 patients with histologic UIP pattern and CTD features and reported no significant deterioration in lung function during 12 months (baseline FVC, 80.6%; 1-year FVC, 78.4%; *p* = 0.48)^22^. Kinder et al. studied 59 patients (30 with IPF and 29 with undifferentiated CTD [UCTD]) and showed that UCTD, rather than IPF, was associated with a substantial improvement in FVC (odds ratio, 8.23; 95% CI, 1.27–53.2; *p* = 0.03) during follow-up^29^. Likewise, Collins et al. studied 124 patients with well-defined ILD and reported that the mean change in DLco over 12 months significantly differed between patients with IPAF and those with IPF (6.3 vs. − 2.9% predicted; *p* < 0.001); moreover, a subgroup analysis among those with UIP in each group showed similar results^30^. In other aspects, the anti-inflammatory treatment group showed a faster decline in lung function than the no-treatment group in the no-IP-hAF group in our study. This finding might be attributed to the harmful effects of anti-inflammatory treatment in patients with IPF^31^.

The IP-hAF group had better survival than the no-IP-hAF group in our study. Previous studies support our results^32,33^. Lim et al. studied 305 patients with ILD (54 with IPAF, 175 with IPF, and 76 with CTD-ILD) and reported that the IPAF group had better survival (mean survival, 73.3 vs. 50.7 months; *p* < 0.001) than the IPF group^32^. Another study on 44 patients with UCTD-UIP and 499 with IPF reported that the UCTD-UIP group showed better survival (median survival, 31 vs. 26 months; *p* = 0.042) than the IPF group^33^. However, others reported contradicting findings^23,34^. Oldham et al. studied 422 patients with IIP or UCTD and showed that patients with IPAF and a UIP pattern (n = 98) had similar survival to those with IPF (n = 268; *p* = 0.51)^23^. Strand et al. studied 321 patients with IPF and 19 with UCTD-UIP and showed no statistical difference in survival between the patients with UCTD-UIP and IPF (median survival, 3.8 vs. 4.4 years; *p* = 0.95)^34^. These contradictory results might be attributed to the difference in the definition of autoimmune features; we used histopathologic findings suggesting CTD for classifying patients with IPF and significant autoimmune features. Our results also suggest that the morphologic domain may further influence the prognosis.

Our study has some limitations. First, a selection bias might exist because we restricted patients to those who underwent surgical lung biopsy at a single centre. However, the clinical features of our patients were comparable to those in previous studies^22,29^. Second, CTD was possibly missed in some of our patients with IIP because minor symptoms and signs could be ignored. However, we used a standardised survey form to identify suspected symptoms and signs of CTD and consulted rheumatologists to determine whether early CTD could be excluded when suspected symptoms and signs were observed. Third, around 80% of patients received steroid therapy. Patients enrolled in this study were diagnosed between February 2000 and November 2007, at which time, steroid therapy was considered as standardized therapy. Although anti-inflammatory treatment has been shown to be harmful in IPF patients, our results suggest that these effects might be different in the IP-hAF group; among the IP-hAF group, changes in lung function were stabilized in the treatment group. Fourth, we excluded those with histopathologic features that should raise concerns about the likelihood of not UIP pattern including prominent lymphoplasmacytic infiltration. Therefore, the whole picture of IP-hAF of UIP pattern is not clear in our study. Finally, we defined the IP-hAF group by using an optimal cut-off level of CTD scores for predicting mortality, instead of the development of CTD, because of the small number of events. However, previous reports suggest that autoimmune features may portend a better prognosis^32,33^. Despite these limitations, our study is potentially the first to investigate the clinical implications of histologic autoimmune features in patients with IPF.

In conclusion, patients with IPF and significant histologic autoimmune features were more frequently women and non-smokers and had more frequent CTD development, more stable lung function, and better prognosis than those without these features. These findings suggest that this group may be classified as a specific subgroup of patients with IPF.

## Supplementary information

Supplementary Information.

Supplementary Figure 1.

Supplementary Figure 2.

Supplementary Figure 3.

Supplementary Figure 4.

Supplementary Figure 5.

Supplementary Figure 6.

Supplementary Figure 7.

Supplementary Figure 8.

Supplementary Figure 9.

## Data Availability

The datasets generated during and/or analysed during the current study are available from the corresponding author on reasonable request.
