# Supplementary material for: Histologic features suggesting connective tissue disease in idiopathic pulmonary fibrosis
Source: Sci Rep. 2020 Dec 3;10:21137. doi: 10.1038/s41598-020-78140-5 (PMC7713371; doi:10.1038/s41598-020-78140-5)
Supplement: Supplementary file 1 — Supplementary Information. [file 41598_2020_78140_MOESM1_ESM.docx]

**Histologic features suggesting connective tissue disease in idiopathic pulmonary fibrosis**

Ho Cheol Kim^1*^, Joon Seon Song^2*^, Sojung Park^1^, Hee-Young Yoon^1^, So Yun Lim^3^, Eun Jin Chae^3^, Se Jin Jang^2^, Jin Woo Song^1^

**Affiliations:**

^1^Department of Pulmonary and Critical Care Medicine, Asan Medical Center, University of Ulsan College of Medicine, Seoul, Republic of Korea

^2^Department of Pathology, Asan Medical Center, University of Ulsan College of Medicine, Seoul, Republic of Korea

^3^Department of Radiology, Asan Medical Center, University of Ulsan College of Medicine, Seoul, Republic of Korea

^*^These authors contributed equally to this work.

**Corresponding author:** Jin Woo Song, MD, PhD

Department of Pulmonary and Critical Care Medicine, University of Ulsan College of Medicine, Asan Medical Center, 88 Olympic-ro 43-gil, Songpa-gu, Seoul 05505, South Korea

Tel: +82-2-3010-3993

Fax: +82-2-3010-6968

E-mail address: jwsongasan@gmail.com

**Table 1.** Predicting factors for survival in patients with IPF assessed using a univariate Cox hazard model

| Variable | HR (95% CI) | *p* value |
| --- | --- | --- |
| Age | 1.012 (0.978-1.046) | 0.502 |
| Male sex | 0.897 (0.568-1.415) | 0.640 |
| Ever-smoker | 1.327 (0.803-2.193) | 0.269 |
| ANA positivity | 0.708 (0.444-1.128) | 0.146 |
| RF positivity | 1.323 (0.679-2.576) | 0.410 |
| CRP | 1.050 (0.630-1.749) | 0.851 |
| FVC | 0.980 (0.966-0.994) | 0.007 |
| DLco | 0.977 (0.963-0.991) | 0.001 |
| TLC | 0.977 (0.961-0.994) | 0.008 |
| Radiologic findings |  |  |
| Reticulation | 2.323 (1.671-3.227) | <0.001 |
| Honeycombing | 1.540 (1.176-2.016) | 0.002 |
| Ground-glass opacity | 0.966 (0.738-1.264) | 0.801 |
| Consolidation | 1.280 (0.719-2.277) | 0.401 |
| Emphysema | 0.899 (0.631-1.281) | 0.556 |
| Traction bronchiectasis | 1.186 (0.941-1.494) | 0.148 |
| Peribronchovascular distribution | 0.809 (0.508-1.289) | 0.373 |
| Subpleural sparing | 0.456 (0.166-1.248) | 0.126 |
| Pleural or pericardial effusion | 1.233 (0.298-5.096) | 0.772 |
| Oesophageal dilatation | 2.151 (0.866-5.343) | 0.099 |
| UIP pattern | 1.785 (1.141-2.791) | 0.011 |
| Pathologic findings |  |  |
| Fibroblastic foci | 1.487 (1.116-1.982) | 0.007 |
| Lymphoid aggregates | 0.859 (0.646-1.144) | 0.298 |
| Plasma cell infiltration | 0.992 (0.769-1.280) | 0.949 |
| Germinal centres | 0.716 (0.539-0.952) | 0.022 |
| Total inflammation | 0.893 (0.654-1.220) | 0.478 |
| Pleural change | 0.930 (0.750-1.154) | 0.511 |
| Organizing pneumonia | 0.890 (0.688-1.150) | 0.373 |
| Intra-alveolar macrophages | 0.994 (0.709-1.394) | 0.972 |
| Honeycombing | 1.120 (0.939-1.335) | 0.207 |
| Stromal fibrosis | 1.097 (0.842-1.428) | 0.492 |
| Perivascular collagen | 1.089 (0.397-2.984) | 0.869 |
| CTD score | 0.921 (0.825-1.028) | 0.142 |
| Treatment with steroids and/or cytotoxic agents | 1.021 (0.538-1.937) | 0.950 |

IPF, idiopathic pulmonary fibrosis; HR, hazard ratio; CI, confidential interval; ANA, anti-nuclear antibody; RF, rheumatoid factor; CRP, C-reactive protein; FVC, forced vital capacity; DLco, diffusing capacity of the lung for carbon monoxide; TLC, total lung capacity; UIP, usual interstitial pneumonia; CTD, connective tissue disease

**Table 2.** Comparison of radiologic findings between the IP-hAF group and no-IP-hAF group among patients with IPF

| Feature | IP-hAF | no-IP-hAF | *p* value |
| --- | --- | --- | --- |
| Patient number | 40 | 74 |  |
| Reticulation | 1.3 ± 0.6 | 1.4 ± 0.6 | 0.429 |
| Honeycombing | 0.7 ± 0.8 | 0.8 ± 0.6 | 0.617 |
| Ground-glass opacity | 0.9 ± 0.9 | 0.7 ± 0.8 | 0.106 |
| Consolidation | 0.3 ± 0.4 | 0.1 ± 0.3 | 0.079 |
| Emphysema | 0.4 ± 0.6 | 0.6 ± 0.6 | 0.192 |
| Traction bronchiectasis | 2.7 ± 0.8 | 2.4 ± 0.9 | 0.172 |
| Peribronchovascular distribution | 19 (47.5) | 22 (30.1) | 0.066 |
| Subpleural sparing | 3 (7.5) | 8 (11.0) | 0.553 |
| Pleural or pericardial effusion | 1 (2.5) | 2 (2.7) | 0.940 |
| Oesophageal dilatation | 1 (2.5) | 4 (5.5) | 0.654 |
| UIP pattern | 25 (62.5) | 54 (73.0) | 0.247 |

Data are presented as mean ± standard deviation or number (%), unless otherwise indicated.

IP-hAF, interstitial pneumonia with histologic autoimmune features; IPF, idiopathic pulmonary fibrosis; UIP, usual interstitial pneumonia

**Table 3** Comparison of histologic findings between the IP-hAF group and no-IP-hAF group among patients with IPF

| Feature | IP-hAF | no-IP-hAF | *p* value |
| --- | --- | --- | --- |
| Patient number | 40 | 74 |  |
| Fibroblastic foci | 1.5 ± 0.7 | 1.5 ± 0.8 | 0.600 |
| Lymphoid aggregates | 2.3 ± 0.5 | 1.2 ± 0.5 | < 0.001 |
| Plasma cell infiltration | 1.8 ± 0.5 | 0.5 ± 0.6 | < 0.001 |
| Germinal centres | 1.1 ± 1.1 | 0.1 ± 0.4 | < 0.001 |
| Total inflammation | 2.2 ± 0.4 | 1.3 ± 0.5 | < 0.001 |
| Pleural change | 2.8 ± 0.7 | 2.2 ± 0.9 | 0.001 |
| Organizing pneumonia | 0.7 ± 0.7 | 0.7 ± 0.9 | 0.986 |
| Intra-alveolar macrophages | 1.3 ± 0.7 | 1.1 ± 0.6 | 0.188 |
| Honeycombing | 2.2 ± 1.0 | 2.7 ± 1.2 | 0.016 |
| Stromal fibrosis | 1.5 ± 0.6 | 1.9 ± 0.8 | 0.012 |
| Perivascular collagen | 0.2 ± 0.4 | 0.1 ± 0.3 | 0.014 |
| CTD score | 5.1 ± 1.4 | 1.8 ± 0.8 | < 0.001 |

Data are presented as mean ± standard deviation or number (%), unless otherwise indicated.

IP-hAF, interstitial pneumonia with histologic autoimmune features; IPF, idiopathic pulmonary fibrosis;

**Table 4.** Predicting factors for IP-hAF in patients with IPF assessed using univariate logistic analysis

| Variable | OR (95% CI) | *p* value | |
| --- | --- | --- | --- |
| Age | 0.988 (0.935-1.044) | 0.667 | |
| Female sex | 5.790 (2.329-14.395) | <0.001 | |
| Ever-smoker | 0.254 (0.110-0.589) | 0.001 | |
| ANA positivity | 0.833 (0.420-1.653) | 0.602 | |
| RF positivity | 0.857 (0.288-2.550) | 0.782 | |
| FVC | 0.981 (0.958-1.005) | 0.126 | |
| DLco | 0.978 (0.955-1.002) | 0.067 | |
| TLC | 0.985 (0.958-1.012) | 0.260 | |
| UIP pattern on HRCT | 0.617 (0.272-1.402) | 0.249 | |
| Reticulation | 0.759 (0.385-1.499) | 0.427 | |
| Honeycombing | 0.861 (0.482-1.540) | 0.614 | |
| Ground-glass opacity | 1.455 (0.916-2.312) | 0.112 | |
| Consolidation | 2.708 (0.971-7.552) | 0.057 | |
| Emphysema | 0.626 (0.310-1.265) | 0.192 | |
| Traction bronchiectasis | 1.398 (0.861-2.271) | 0.175 | |
| Peribronchovascular distribution | 2.097 (0.945-4.653) | 0.068 | |
| Subpleural sparing | 0.659 (0.165-2.637) | 0.555 | |
| Pleural or pericardial effusion | 0.910 (0.080-10.361) | 0.940 |  |
| Esophageal dilatation | 0.442 (0.048-4.098) | 0.473 |  |

IP-hAF, interstitial pneumonia with histologic autoimmune features; IPF, idiopathic pulmonary fibrosis; OR, odds ratio; CI, confidential interval; ANA, anti-nuclear antibody; RF, rheumatoid factor; FVC, forced vital capacity; DLco, diffusing capacity of the lung for carbon monoxide; TLC, total lung capacity; UIP, usual interstitial pneumonia; HRCT, high-resolution computed tomography

**Table 5.** Changes in lung function during 12 months after diagnosis

| Lung function | Group | Mean changes from 0 to 6 months | *p* value^*^ | *p* value^**^ | Mean changes from 0 to 12 months | *p* value^*^ | *p* value^**^ |
| --- | --- | --- | --- | --- | --- | --- | --- |
| FVC, % predicted | Total | -2.88 ± 10.04 | 0.013 |  | -6.28 ± 12.93 | <0.001 |  |
|  | IP-hAF group | -0.26 ± 10.20 | 0.889 | 0.060 | -2.00 ± 13.93 | 0.471 | 0.028 |
|  | No-IP-hAF group | -4.62 ± 9.65 | 0.002 |  | -9.13 ± 11.54 | <0.001 |  |
| DLco, % predicted | Total | -3.28 ± 18.31 | 0.118 |  | -5.84 ± 15.20 | 0.004 |  |
|  | IP-hAF group | 2.68 ± 22.06 | 0.504 | 0.019 | 0.44 ± 11.38 | 0.848 | 0.006 |
|  | No-IP-hAF group | -7.21 ± 14.30 | 0.001 |  | -10.08 ± 16.10 | 0.001 |  |
| TLC, % predicted | Total | -3.27 ± 8.84 | 0.002 |  | -4.44 ± 10.17 | 0.001 |  |
|  | IP-hAF group | -1.47 ± 8.86 | 0.372 | 0.151 | -1.04 ± 10.66 | 0.624 | 0.023 |
|  | No-IP-hAF group | -4.47 ± 8.72 | 0.001 |  | -6.97 ± 9.14 | <0.001 |  |

Data are presented as mean ± standard deviation.

FVC, forced vital capacity; DLco, diffusing capacity of the lung for carbon monoxide; TLC, total lung capacity; IP-hAF, interstitial pneumonia with histologic autoimmune features

^*^*p* value indicates changes over time in the same group (paired t-test), and ^**^*p* value indicates a difference between the IP-hAF and no-IP-hAF groups (Student’s t-test).

**Table 6.** Changes in lung function during 12 months after diagnosis according to treatment

| Lung function | Group | Mean changes from 0 to 6 months | *p* value^*^ | *p* value^**^ | Mean changes from 0 to 12 months | *p* value^*^ | *p* value^**^ |
| --- | --- | --- | --- | --- | --- | --- | --- |
| IP-hAF group | | | | | | | |
| FVC, % predicted | Tx group | 0.35 ± 8.88 | 0.844 | 0.462 | -1.18 ± 14.66 | 0.709 | 0.494 |
|  | No-Tx group | -3.40 ± 16.53 | 0.669 |  | -6.50 ± 8.89 | 0.240 |  |
| DLco, % predicted | Tx group | 4.85 ± 23.17 | 0.296 | 0.218 | 1.57 ± 11.12 | 0.525 | 0.264 |
|  | No-Tx group | -8.60 ± 10.21 | 0.133 |  | -5.50 ± 12.50 | 0.444 |  |
| TLC, % predicted | Tx group | -0.80 ± 9.13 | 0.665 | 0.366 | -0.41 ± 11.44 | 0.868 | 0.187 |
|  | No-Tx group | -4.80 ± 7.19 | 0.210 |  | -4.50 ± 3.42 | 0.078 |  |
| No-IP-hAF group | | | | | | | |
| FVC, % predicted | Tx group | -4.10± 9.63 | 0.011 | 0.426 | -8.45 ± 11.91 | <.001 | 0.400 |
|  | No-Tx group | -7.13 ± 9.98 | 0.083 |  | -12.83 ± 9.22 | 0.019 |  |
| DLco, % predicted | Tx group | -9.00 ± 12.91 | <0.001 | 0.058 | -11.35 ± 13.98 | <0.001 | 0.280 |
|  | No-Tx group | 1.50 ± 18.32 | 0.824 |  | -3.50 ± 25.15 | 0. 747 |  |
| TLC, % predicted | Tx group | -4.50 ± 8.76 | 0.004 | 0.960 | -6.45 ± 8.75 | <0.001 | 0.356 |
|  | No-Tx group | -4.33 ± 9.07 | 0.190 |  | -11.00 ± 12.49 | 0.176 |  |

Data are presented as mean ± standard deviation.

FVC, forced vital capacity; DLco, diffusing capacity of the lung for carbon monoxide; TLC, total lung capacity; IP-hAF, interstitial pneumonia with histologic autoimmune features; Tx, treatment

^*^*p* value indicates changes over time in the same group (paired t-test), and ^**^*p* value indicates a difference between the Tx and no-Tx groups (Student’s t-test).

**Table 7.** Predicting factors for survival in patients with IPF assessed using a univariate Cox hazard model

| Variable | HR (95% CI) | *p* value |
| --- | --- | --- |
| Age | 1.012 (0.978-1.046) | 0.502 |
| Male sex | 0.897 (0.568-1.415) | 0.640 |
| Ever-smoker | 1.327 (0.803-2.193) | 0.269 |
| ANA positivity | 0.708 (0.444-1.128) | 0.146 |
| RF positivity | 1.323 (0.679-2.576) | 0.410 |
| CRP | 1.050 (0.630-1.749) | 0.851 |
| FVC | 0.980 (0.966-0.994) | 0.007 |
| DLco | 0.977 (0.963-0.991) | 0.001 |
| TLC | 0.977 (0.961-0.994) | 0.008 |
| Radiologic findings |  |  |
| Reticulation | 2.323 (1.671-3.227) | <0.001 |
| Honeycombing | 1.540 (1.176-2.016) | 0.002 |
| Ground-glass opacity | 0.966 (0.738-1.264) | 0.801 |
| Consolidation | 1.280 (0.719-2.277) | 0.401 |
| Emphysema | 0.899 (0.631-1.281) | 0.556 |
| Traction bronchiectasis | 1.186 (0.941-1.494) | 0.148 |
| Peribronchovascular distribution | 0.809 (0.508-1.289) | 0.373 |
| Subpleural sparing | 0.456 (0.166-1.248) | 0.126 |
| Pleural or pericardial effusion | 1.233 (0.298-5.096) | 0.772 |
| Esophageal dilatation | 2.151 (0.866-5.343) | 0.099 |
| UIP pattern | 1.785 (1.141-2.791) | 0.011 |
| Pathologic findings |  |  |
| Fibroblastic foci | 1.487 (1.116-1.982) | 0.007 |
| Lymphoid aggregates | 0.859 (0.646-1.144) | 0.298 |
| Plasma cell infiltration | 0.992 (0.769-1.280) | 0.949 |
| Germinal centres | 0.716 (0.539-0.952) | 0.022 |
| Total inflammation | 0.893 (0.654-1.220) | 0.478 |
| Pleural change | 0.930 (0.750-1.154) | 0.511 |
| Organizing pneumonia | 0.890 (0.688-1.150) | 0.373 |
| Intra-alveolar macrophages | 0.994 (0.709-1.394) | 0.972 |
| Honeycombing | 1.120 (0.939-1.335) | 0.207 |
| Stromal fibrosis | 1.097 (0.842-1.428) | 0.492 |
| Perivascular collagen | 1.089 (0.397-2.984) | 0.869 |
| IP-hAF group | 0.743 (0.485-1.139) | 0.172 |
| Treatment with steroids and/or cytotoxic agents | 1.021 (0.538-1.937) | 0.950 |

IPF, idiopathic pulmonary fibrosis; HR, hazard ratio; CI, confidential interval; ANA, anti-nuclear antibody; RF, rheumatoid factor; CRP, C-reactive protein; FVC, forced vital capacity; DLco, diffusing capacity of the lung for carbon monoxide; TLC, total lung capacity; UIP, usual interstitial pneumonia; IP-hAF, interstitial pneumonia with histologic autoimmune features

**Supplementary figure legends**

**Figure 1.** Histologic grade of fibroblastic foci. (A) mild (1 point) represents one or two fibroblastic foci, (B) moderate ( 2 point) represents occasional fibroblastic foci and (C) marked (3 point) shows frequent fibroblastic foci.(magnification, 12.5x, H&E)

**Figure 2.** Histologic grade of plasma cell infiltration. The scores are made by semiquantatively. (A) score 0 represents absence of plasma cells. (B) score 1 represents minimal plasma cell infiltration (less than 10 cells/ high power field). (C) score 2 represents moderate and occasional plasma cells are present. (D) score 3 represents marked plasma cell infiltration. (magnification, 400x, H&E)

**Figure 3.** Histologic grade of germinal center. Germinal centers (GCs) count up to three consecutive fields with the lowest magnification and the average of three fields are calculated. (A) score 1 represents minimal with one or two germinal centers. (B) score 2 represents less than 10 GCs. (C) score 3 represents more than 10 GCs (magnification, 20 x, H&E)

**Figure 4.** Histologic grade of total inflammation. The amount of all inflammatory cells including lymphocyte, plasma cells and macrophages are scored semiquantitively. The inflammation scores three tiers; (A) 1, mild; (B) 2, moderate and (C) 3, marked. (magnification, 20x, H&E)

**Figure 5.** Histologic grade of pleural change. Pleural change is divided four categories; (A) score 1, no change; (B) score 2, pleural fibrosis; (C) score 3, fibrinous pleural change; and (D) score 4, both pleuritis and fibrosis. (magnification, 20x, H&E)

**Figure 6.** Histologic grade of organizing pneumonia. The scores are graded according to the amount of intra-alveolar fibroblastic plugs. (A) score 0, no; (B) score 1, mild; (C) score 2, moderate; and (D) score 3, marked. (magnification, 40x, H&E)

**Figure 7.** Histologic grade of honeycombing. Honeycombing is scored according to the measured size of the largest honeycombing spaces: score 0, none; 1, <1 mm; 2, 1-3 mm; 3, 3-5 mm; and 4, >5 mm. (A) The size measures 1.63 mm, scoring as 2 and (B) the size measures 4.76 mm, scoring as 3. (magnification, 100x, H&E)

**Figure 8.** Representative images of histologic characteristics. a) patient with typical UIP, b) patient with IP-hAF, c) patient with CTD

UIP, usual interstitial pneumonia; IP-hAF, interstitial pneumonia with histologic autoimmune features; CTD: connective tissue disease

**Figure 9.** Changes in lung function during 12 months after diagnosis between the treatment and no-treatment groups of patients with IPF. a) Changes in FVC in patients with IP-hAF, b) Changes in DLco in patients with IP-hAF, c) Changes in TLC in patients with IP-hAF, d) Changes in FVC in patients with no-IP-hAF, e) Changes in DLco in patients with no-IP-hAF, f) Changes in TLC in patients with no-IP-hAF. Each symbol with error bars represents the mean and standard deviation.

IPF, idiopathic pulmonary fibrosis; IP-hAF, interstitial pneumonia with histologic autoimmune features; FVC, forced vital capacity; DLco, diffusing capacity of the lung for carbon monoxide; TLC, total lung capacity; Tx, treatment; No-Tx, No treatment.
