# Supplementary figures and images for: Histologic features suggesting connective tissue disease in idiopathic pulmonary fibrosis
Source: Sci Rep. 2020 Dec 3;10:21137. doi: 10.1038/s41598-020-78140-5 (PMC7713371; doi:10.1038/s41598-020-78140-5)

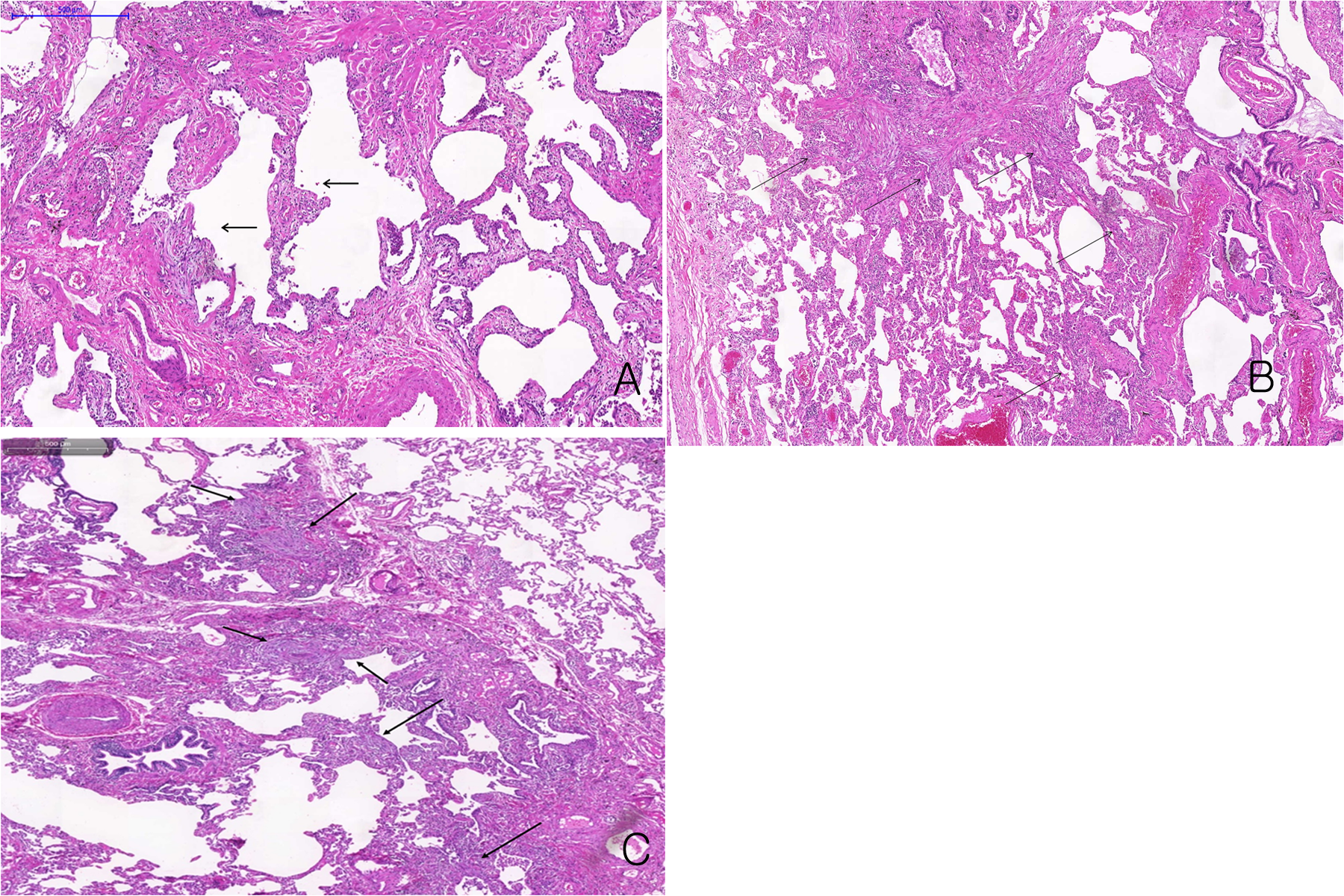

Supplement: Supplementary file 2 — Supplementary Figure 1. [file 41598_2020_78140_MOESM2_ESM.tif]

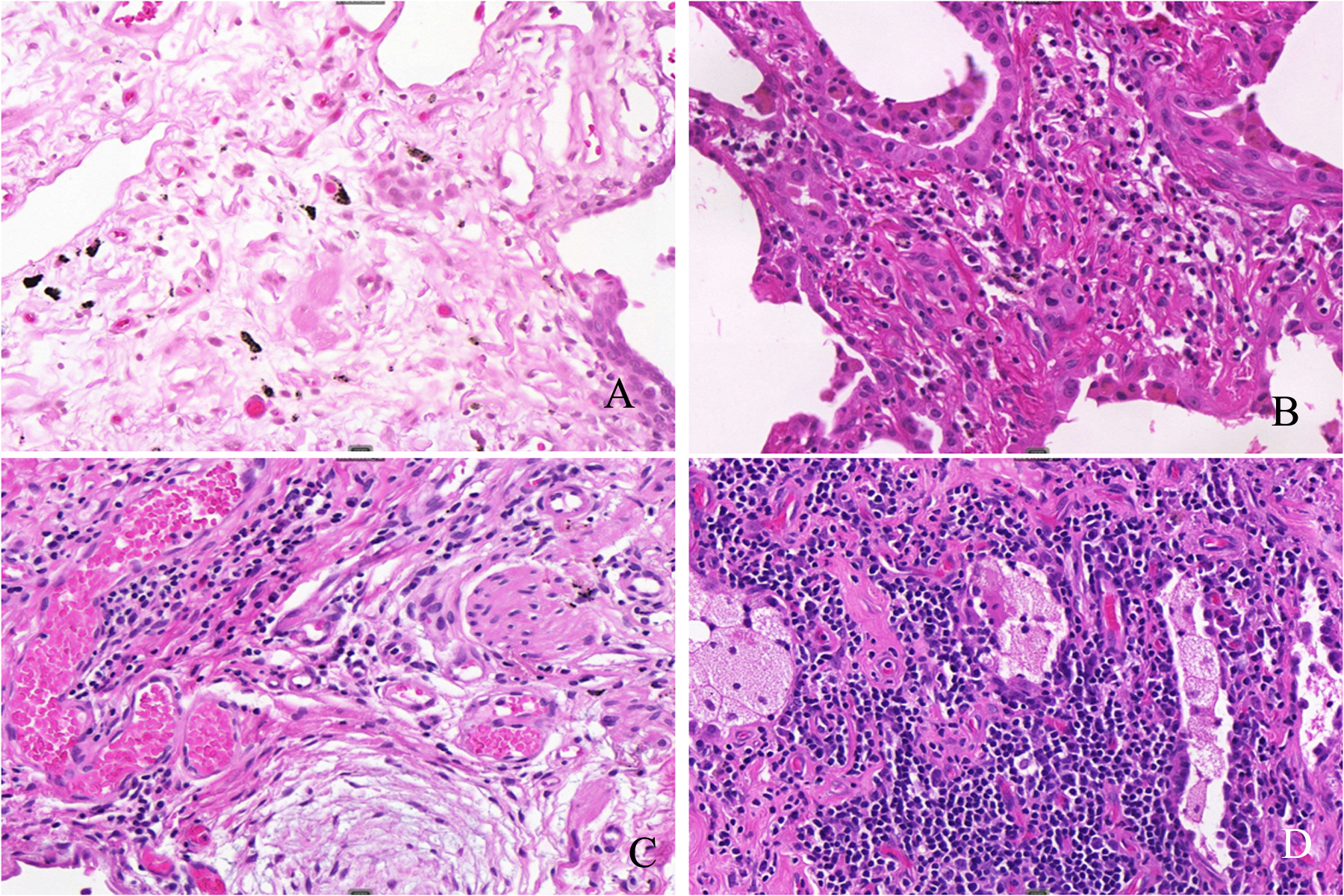

Supplement: Supplementary file 3 — Supplementary Figure 2. [file 41598_2020_78140_MOESM3_ESM.tif]

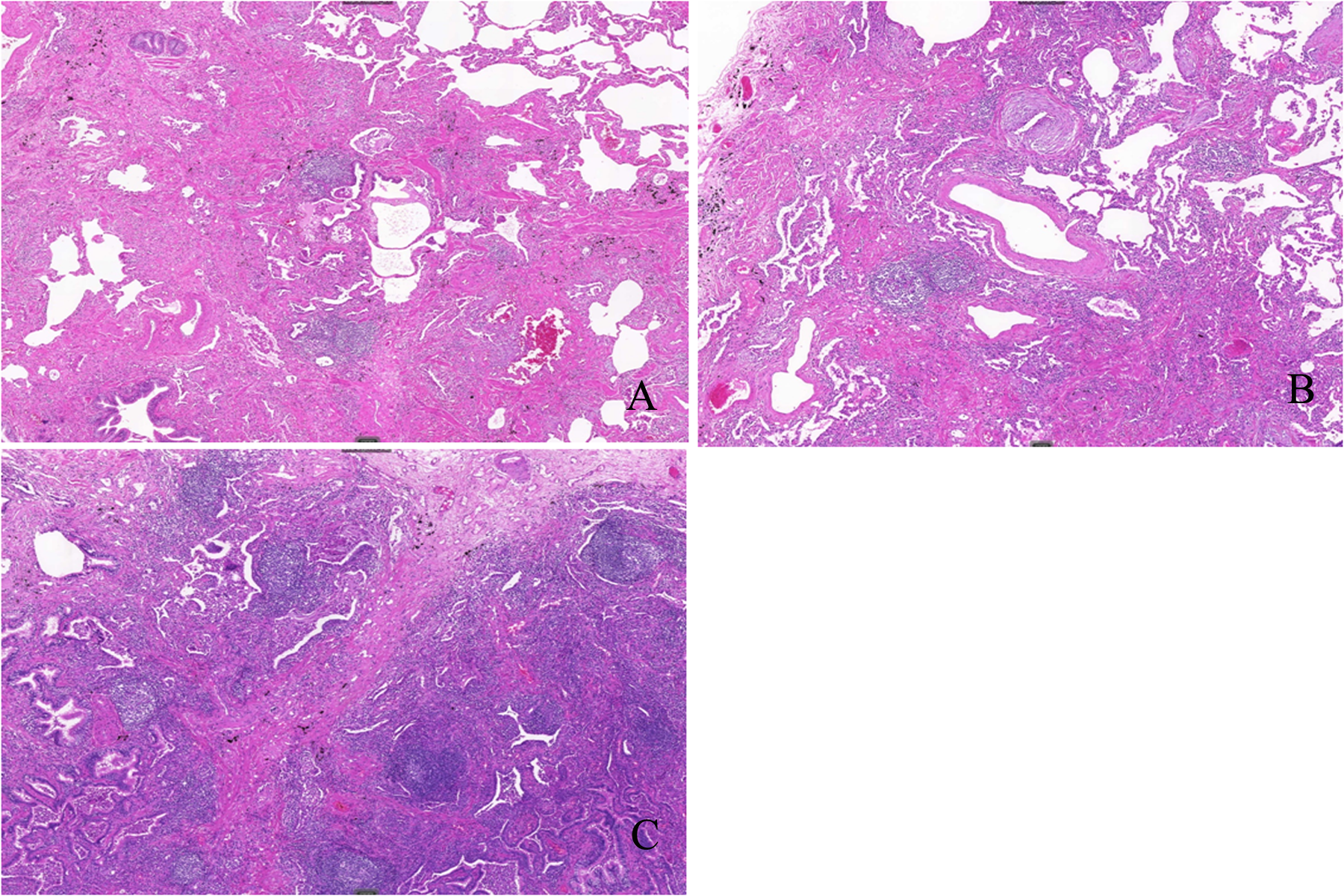

Supplement: Supplementary file 4 — Supplementary Figure 3. [file 41598_2020_78140_MOESM4_ESM.tif]

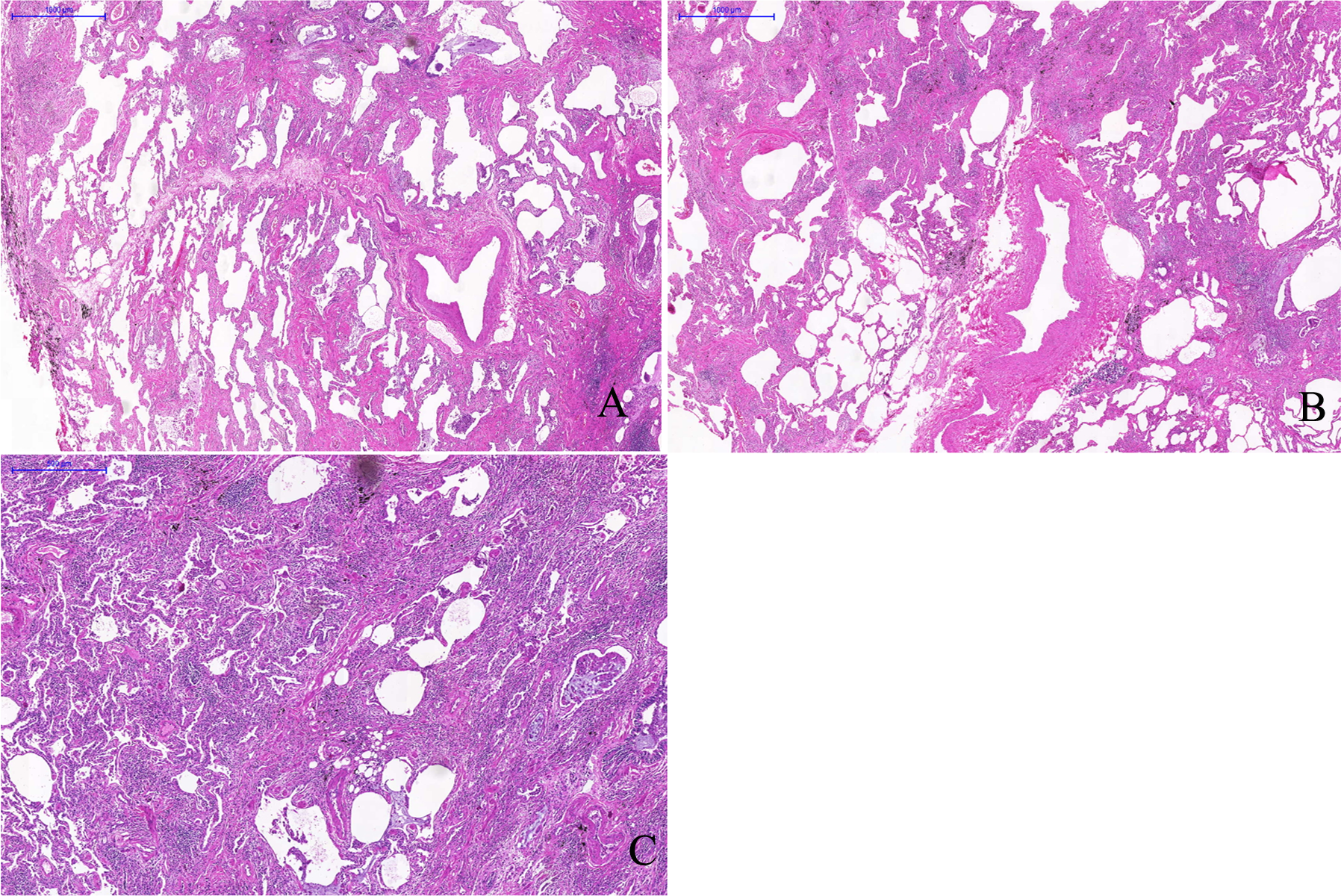

Supplement: Supplementary file 5 — Supplementary Figure 4. [file 41598_2020_78140_MOESM5_ESM.tif]

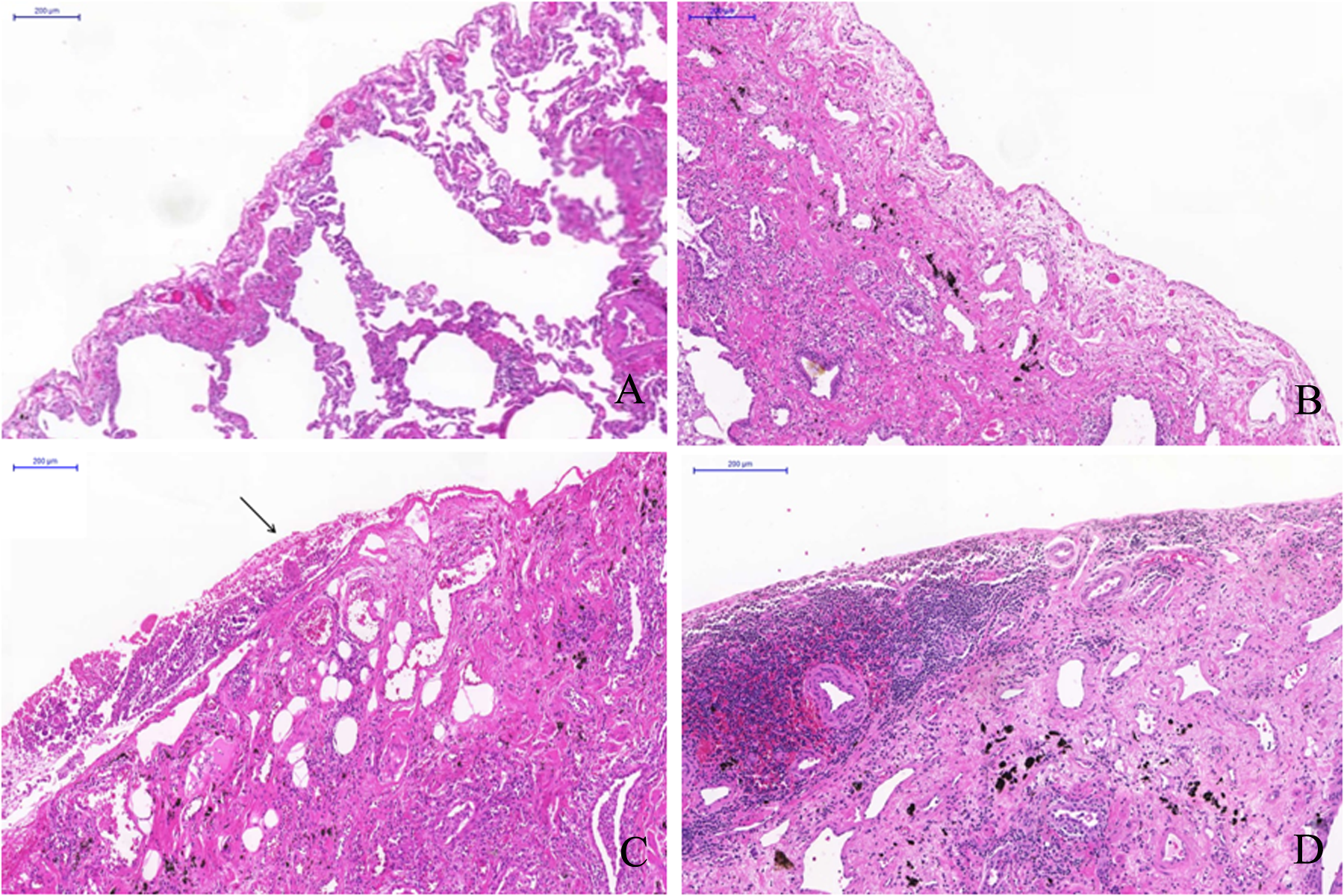

Supplement: Supplementary file 6 — Supplementary Figure 5. [file 41598_2020_78140_MOESM6_ESM.tif]

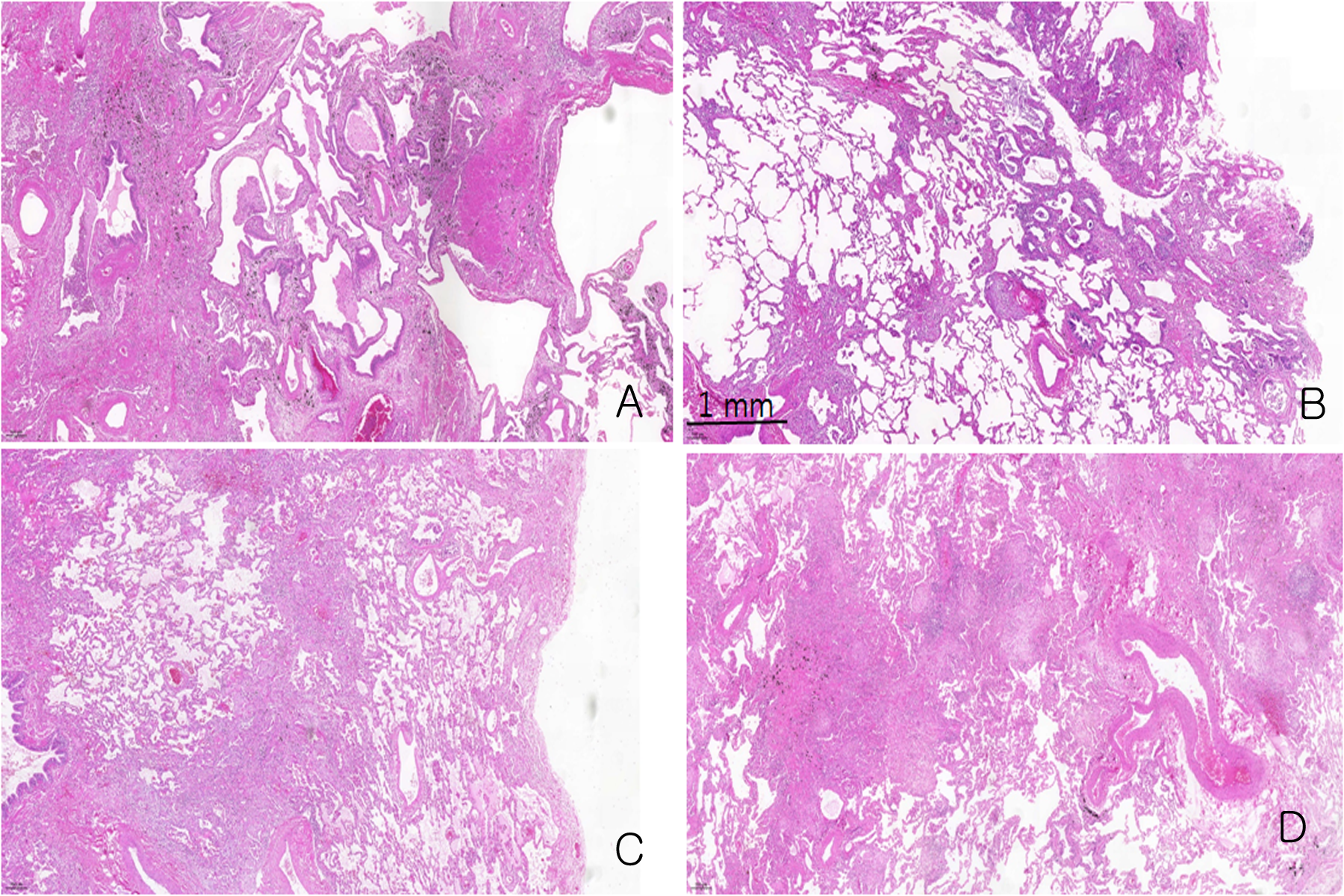

Supplement: Supplementary file 7 — Supplementary Figure 6. [file 41598_2020_78140_MOESM7_ESM.tif]

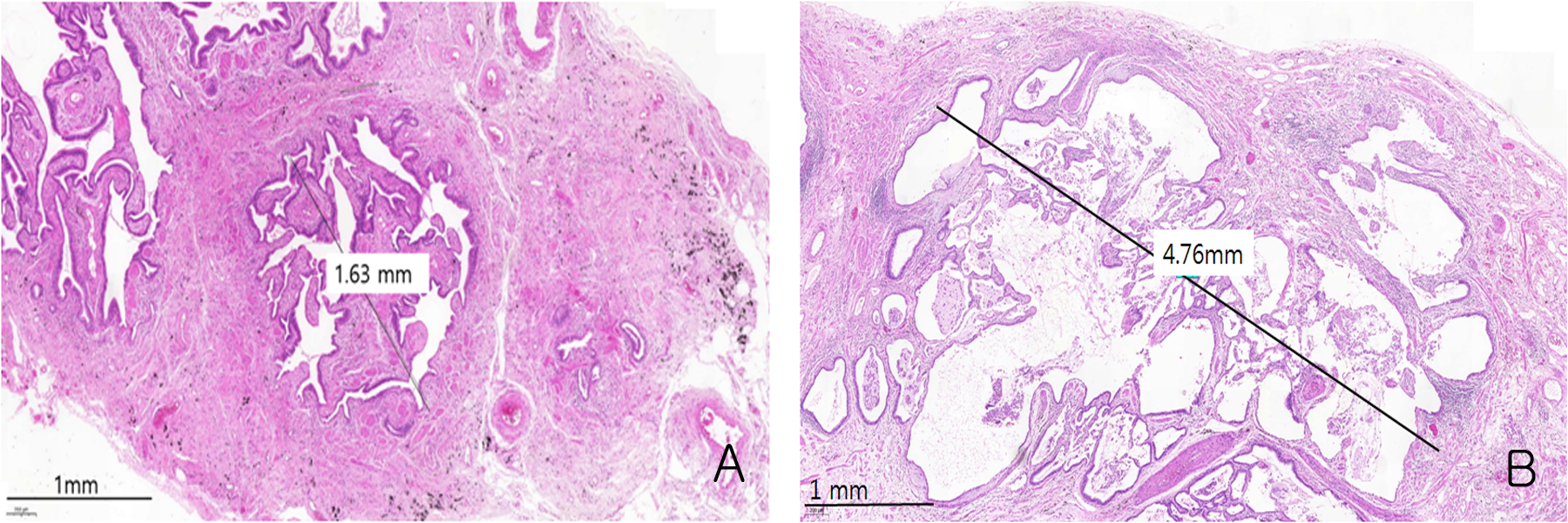

Supplement: Supplementary file 8 — Supplementary Figure 7. [file 41598_2020_78140_MOESM8_ESM.tif]

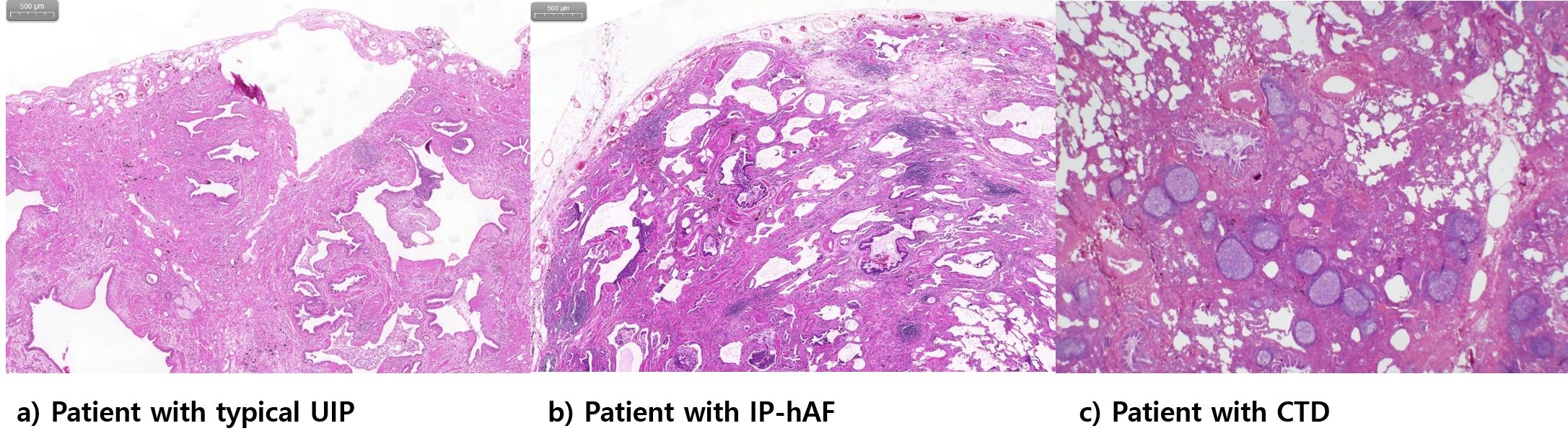

Supplement: Supplementary file 9 — Supplementary Figure 8. [file 41598_2020_78140_MOESM9_ESM.tif]

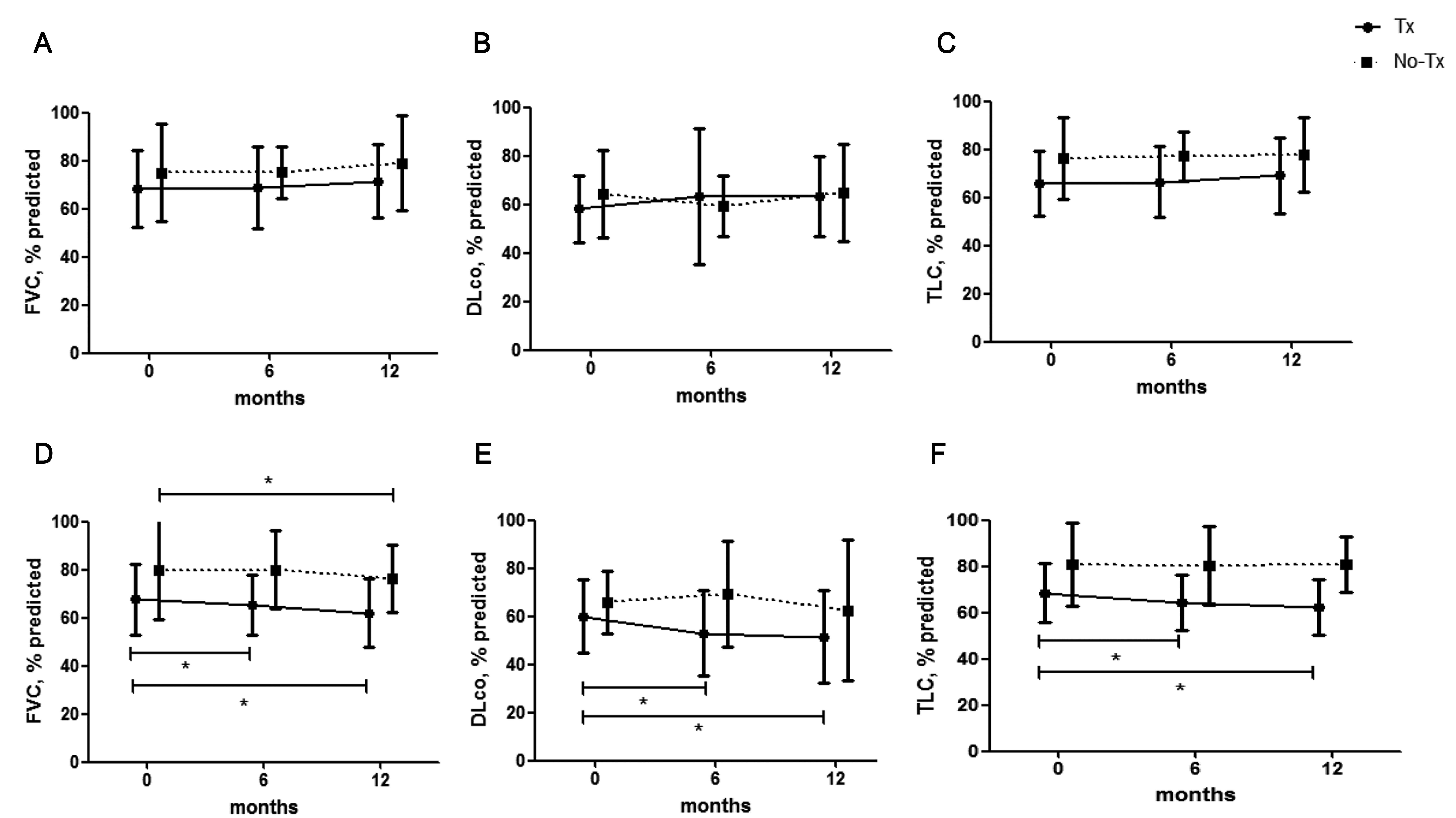

Supplement: Supplementary file 10 — Supplementary Figure 9. [file 41598_2020_78140_MOESM10_ESM.tif]
